# Postural Sway and Gaze Can Track the Complex Motion of a Visual Target

**DOI:** 10.1371/journal.pone.0119828

**Published:** 2015-03-16

**Authors:** Vassilia Hatzitaki, Nicholas Stergiou, George Sofianidis, Anastasia Kyvelidou

**Affiliations:** 1 Motor Control and Learning Laboratory, School of Physical Education and Sport Science, Aristotle University of Thessaloniki, Thessaloniki, Greece; 2 Biomechanics Research Building, University of Nebraska at Omaha, Omaha, Nebraska, United States of America; UMR8194, FRANCE

## Abstract

Variability is an inherent and important feature of human movement. This variability has form exhibiting a chaotic structure. Visual feedback training using regular predictive visual target motions does not take into account this essential characteristic of the human movement, and may result in task specific learning and loss of visuo-motor adaptability. In this study, we asked how well healthy young adults can track visual target cues of varying degree of complexity during whole-body swaying in the Anterior-Posterior (AP) and Medio-Lateral (ML) direction. Participants were asked to track three visual target motions: a complex (Lorenz attractor), a noise (brown) and a periodic (sine) moving target while receiving online visual feedback about their performance. Postural sway, gaze and target motion were synchronously recorded and the degree of force-target and gaze-target coupling was quantified using spectral coherence and Cross-Approximate entropy. Analysis revealed that both force-target and gaze-target coupling was sensitive to the complexity of the visual stimuli motions. Postural sway showed a higher degree of coherence with the Lorenz attractor than the brown noise or sinusoidal stimulus motion. Similarly, gaze was more synchronous with the Lorenz attractor than the brown noise and sinusoidal stimulus motion. These results were similar regardless of whether tracking was performed in the AP or ML direction. Based on the theoretical model of optimal movement variability tracking of a complex signal may provide a better stimulus to improve visuo-motor adaptation and learning in postural control.

## Introduction

Intentional postural tracking of visual cues is used in balance assessment [1] and rehabilitation [2] for improving visuo-motor integration through a recalibration of the sensory systems contributing to postural control [3]. The amount and type of visual information provided in the target and feedback signals determines how visuo-motor learning generalizes to other sensory-motor tasks [4]. One major concern is that the regularity of visual target motions used to guide tracking performance evokes the use of predictive mechanisms [5] with limited access to sensory-motor recalibration (perception-action) processes. It is therefore possible that an internal plan, developed through practice, controls the motor output with no need to attend to the target motion anymore, which may explain why this type of visuo-motor learning is task specific. The specificity of visuo-motor learning together with the re-organization of posture into a single degree of freedom control strategy [6] may limit the number of functional coordination solutions the motor system uses in order to adapt to novel tasks or environmental constraints.

Healthy movement behavior on the other hand assumes the maintenance of some degree of intrinsic variability and flexibility in the motor output. Optimal movement variability is associated with complex interactions across multiple control systems, feedback loops and regulatory processes that enable an organism to function and adapt to the demands of everyday life [7,8]. It has been suggested that the structure of these flexible multi-scale interactions resembles mathematical chaos [7,8]. In fact recent literature from several disciplines and medical areas, including brain function and disease dynamics have shown that many apparently “noisy” phenomena are the result of nonlinear interactions and have deterministic origins. Thus, complexity in human movement is organized and could be described as highly variable fluctuations in physiological processes resembling mathematical chaos [9]. This physiological organized complexity is recognized as an inherent attribute of healthy biological systems, whereas its loss due to pathology or aging is thought to reduce the adaptive capabilities of the individual [10,11]. A loss of this complexity can refer to either an overly constrained, periodic system, or an overly random, incoherent system. Thus, healthy human function requires the coexistence of the oppositional factors of coherence and chaos.

Based on this theoretical paradigm, visuo-motor learning should allow the system’s intrinsic variability to be maintained or even exploited. One way to attain this goal in postural tracking practice is to introduce visual cues of varying degree of organized complexity. However, one question that needs to be addressed first is how well humans can track with their gaze and posture a visual target that moves in an unpredictable, chaotic pattern. Evidence from auditory synchronization research introduced the notion of strong anticipation as a new framework for explaining the coordination of tapping behavior with chaotic signals [12]. In this context, tapping is sensitive to the non-local temporal structure of environmental (chaotic) stimuli whereas an internal cognitive model is not viable anymore to explain synchronization to unpredictable chaotic auditory cues [13].

In a recent study examining the dynamic response of posture and gaze to visual target motions of varying complexity [14], gaze was particularly sensitive to the complexity of the visual target motion whereas posture remained relatively unaffected. Spontaneous looking while standing however may not be a sufficiently challenging task for the visual regulation of stance as opposed to intentional tracking of a visual target, which requires the modulation of posture according to the visual stimulus motion information. In the latter case, the coupling between visual motion stimuli of different frequency and the body-head movement is stronger when compared to spontaneous unconscious looking [15]. When postural tracking of a predictable and an unpredictable visual target motion was compared [1], performers tracked the predictable target motion with a shorter delay and higher accuracy than the unpredictable target motion. This may be explained by the fact that humans tend to rely on the stationary properties of the target signal rather than the less stationary performance feedback signal for controlling postural sway [4,16]. The target motion used in this study however was completely random. It is still not known what visual stimulus parameters/characteristics along the spectrum of complexity regarding the structure motion can optimize the perception, processing and integration of visual information in order to control posture.

The aim of the present study was to examine how well healthy adults can track (with their whole body) visual target cues that differ in the degree of complexity (periodic, chaotic, noise) during voluntary visually guided sway in the Anterior-Posterior (A/P) and Medio-Lateral (M/L) direction. This was reflected in the degree of target-performance (posture and gaze) coupling assessed using both linear (coherence) and non-linear metrics (Cross-Approximate entropy). We hypothesized that participants would entrain their posture and gaze better to the chaotic rather than the noise or periodic visual target motion because chaos is an inherent feature of biological motion.

## Methods

### Participants

Ten (10) healthy young male adults (age: 23.5±3.5 years, height: 175.4 ± 4.3 cm, mass: 79.6 ± 7.43kg), recruited from the university students, volunteered to participate in this study. All participants were free from any neurological or musculoskeletal impairment and had normal or corrected to normal vision. Participants gave their informed consent prior to their inclusion in the study. The experiment was performed with the approval of Aristotle University’s ethics committee on human research in accordance with the Declaration of Helsinki. Participants gave their written informed consent prior to their inclusion in the study.

### Apparatus, Task and Stimuli

**Fig 1:**
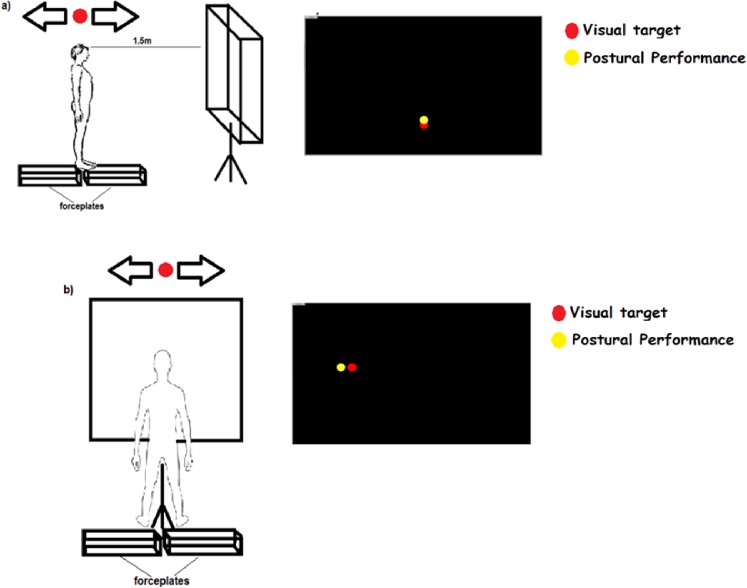
Set up. Experimental setup and visual stimuli presentation a: Medio_Lateral direction (ML), b: Anterior-Posterior (AP) direction

Two adjacent force plates (Balance Plate 6501, Bertec, USA) recorded the ground reaction forces at 100 Hz during experimental task performance. The resultant vertical ground reaction force normalized to the participant’s Body Weight (BW) was displayed as a yellow dot in real-time via an overhead projector (View Sonic, PJ510) on a large projection screen (2.2m horizontal x 1.6m vertical) located in front of the standing participant at a distance of 1.5 m, at eyelevel (Fig. 1). The force feedback signal was superimposed on the computer simulated target signal displayed as a red dot (Fig. 1). An eye tracking system (Dikablis, Ergoneers) tracked the gaze of the standing subject at 50 Hz during task performance. Force, gaze and target motions were synchronously sampled (100 Hz) and digitized through a Vicon system’s (Vicon Motion Systems, Oxford, UK) data acquisition board (MX Giganet).

Posture and gaze data were collected in a single experimental session at the Motor Control and Learning laboratory of Aristotle University of Thessaloniki, Greece. At the starting position (Fig. 1), the participant stood over the midline of the two force platforms distributing his BW evenly between the platforms while maintaining a normal stance position (inter-malleolar distance was set at 10 cm). The experimental task required tracking of the moving target (i.e. red dot) by shifting BW (displayed by the yellow dot) between the two platforms in the Antero-Posterior (AP) or Medio-Lateral (ML) direction. The instruction was to use the yellow dot in order to follow the movement of the red dot as closely as possible. The red target moved either in the up-down (Fig. 1A) direction resulting in Anterior-Posterior (AP) body sway or sideways (Fig. 1B) resulting in Medio-Lateral (ML) sway. Target tracking in the AP direction required an additional visuo-motor transformation of the target’s screen (up-down) into the body’s spatial (forward-backward) coordinate system. A perceptual bias for up-far and down-close is reported for vertical saccadic eye movements [17] as well as depth perception of visual surfaces [18]. In our experiment, participants were free to select their intuitive sway-target direction matching in the AP tracking task. In accordance with the perceptual bias hypothesis, all participants intuitively swayed forward in order to track the upward target motion (perceived as moving away) and swayed backward when tracking the downward target motion (perceived as an approaching motion). Feet position on the platforms was adjusted based on sway direction (Fig. 1A). Participants were required to track three different target motions (periodic, chaotic, brown noise) in the AP and ML direction which resulted in 6 experimental trials that were fully counterbalanced to account for order effects. Each trial lasted 120 s.

Target signals were constructed using custom Matlab (Version 7.9, MathWorks Inc, USA) algorithms while the data series were accessed and displayed through the main Labview (version 8.6, National Instruments Corporation, 2008) application onto the monitor. The maximum peak-to-peak amplitude of the target signals was scaled to 90% of BW in both directions. Each signal comprised 6000 data points. The position of the stimulus on the monitor was updated at a rate of 50 Hz providing 120 seconds of continuous target stimulus motion. All target signals had the same median frequency (0.25 Hz) that corresponds to the dominant frequency of self paced sway as this was estimated by pilot tests and prior experiments [19]. This resulted in 30 full sway cycles performed in 120 s.

**Fig 2:**
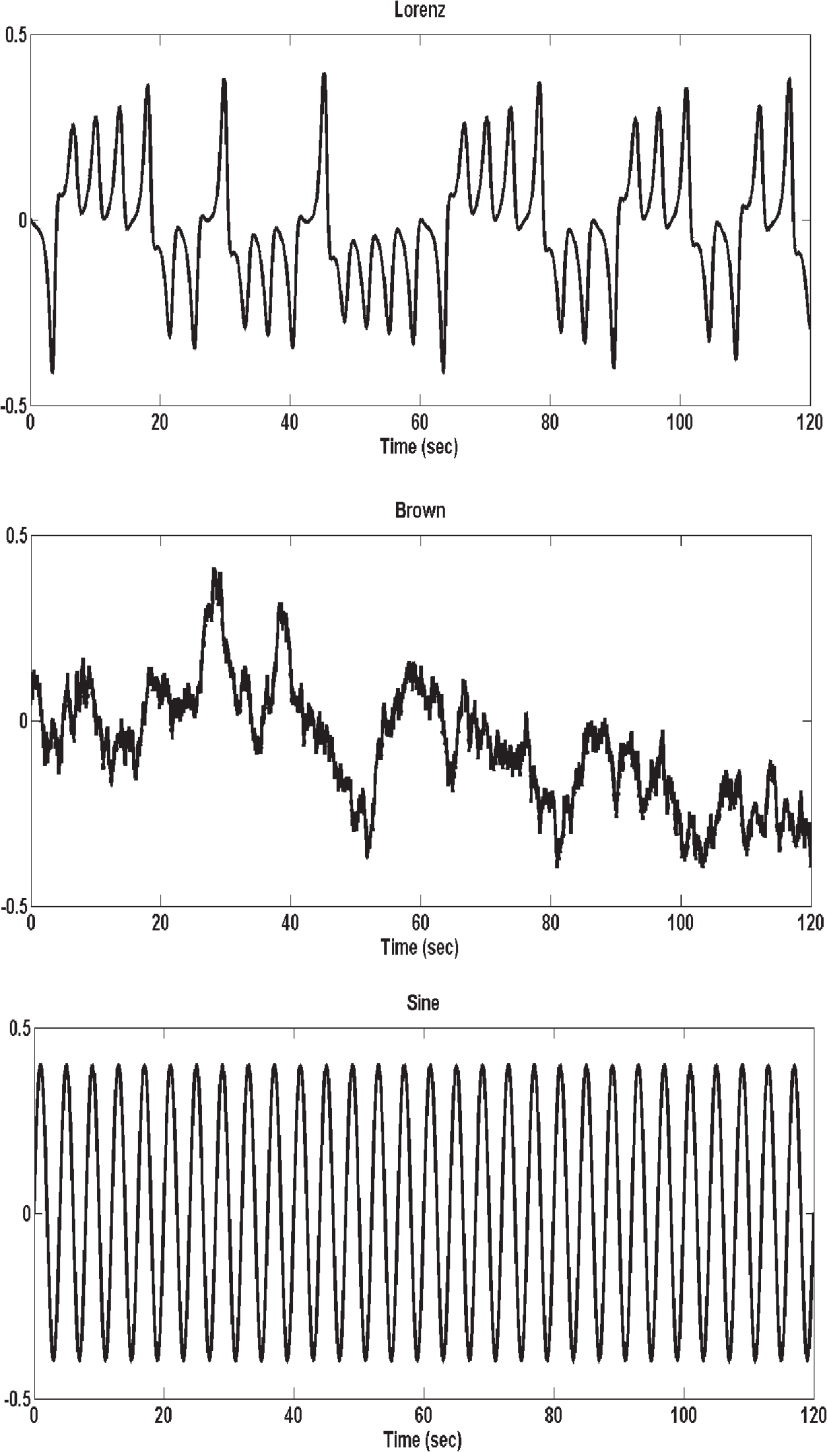
Stimulus signals. Signals used for constructing stimuli motion: Lorenz attractor (a), brown noise (b) and sine (c)

Three signals of different degree of complexity (Fig. 2) were used to construct the frequency structure of the motion of the visual target signal (red dot): a sine wave, a chaotic signal generated using a Lorenz attractor and a brown noise signal. These specific signals were chosen, as they span the spectrum of signal properties related to the aims of the current investigation. The sine wave signal was created using the sin function [sine = sin (2*pi*f/fs*t)] in Matlab. This signal represents simple periodic redundancy, similar to what would be seen from a frictionless clock pendulum. The Lorenz signal was generated by fixing the following parameters at: σ = 10, β = 8/3 and r = 28 and the initial conditions: x0 = 0.1, y0 = 0.1 and z0 = 0.1. The signal characteristics were: h (time resolution) = 0.0040, steps (number of points) = 10000 (we choose 6000 data points from y axis [y (4000:10000)], noise flag = 0. The Lorenz signal as a model closely resembles a double-pendulum, which has previously been shown to emulate the dynamics of human posture [20]. The brown noise signal was created using readily available software (http://people.sc.fsu.edu/~jburkardt/m_src/brownian_motion_simulation/brownian_motion_simulation.m) with input, integers M (the spatial dimension) = 2, N (the number of time steps) = 10000 [we choose 6000 data points (4000:10000)], the d (diffusion coefficient) = 10.0 and the real T (the total time) = 1.0.This signal was selected as it provides an non-periodic motion structure, but also affords continuous smooth pursuit eye movement responses. Furthermore, work by Collins and Deluca [21], have suggested that human posture can be modeled as Brownian motion.

### Data Analysis

In order to compare how well one’s postural sway and gaze was coupled to the visual target motions two different metrics of coupling were calculated in Matlab (Matlab7.9, MathworksInc, USA): the spectral coherence (Coh) and Cross Approximate Entropy (CrossApEn). These two measures estimate the magnitude and the temporal structure of the association respectively between a) the resultant force vector and the target stimulus motion b) the gaze (eye coordinate in the respective target’s direction) and the target stimulus motion in the AP and ML directions.

Spectral coherence measures the degree of correlation between two signals in the frequency domain. The magnitude-squared coherence is estimated as a function of sway frequency with values between 0 and 1 that indicate how well force and gaze couple to the target stimulus motion at the dominant sway frequency of 0.25 Hz [19]. Cross-ApEn quantifies the regularity of patterns in a pair of related time series [22] and is indicative of the dimensionality of control of the two signals [23]. Cross-ApEn has the advantage of being independent of the variance of the signals under comparison. The calculation of Cross-ApEn is similar to approximate entropy (ApEn) with the exception that successive two-point vectors of one signal are compared with current and previous two-point vectors of another signal [22]. Larger Cross-ApEn values indicate greater joint signal asynchrony while lower Cross-ApEn values indicate greater joint signal synchrony.

The effect of direction and target stimulus motion on the performance-target coupling was evaluated employing a 2 (direction) x 3 (stimulus complexity) repeated measures ANOVA. Significant interactions between factor levels were further analyzed by performing pairwise (t-tests) comparisons between the respective factor levels after adjusting p values for multiple comparisons.

## Results

**Fig 3:**
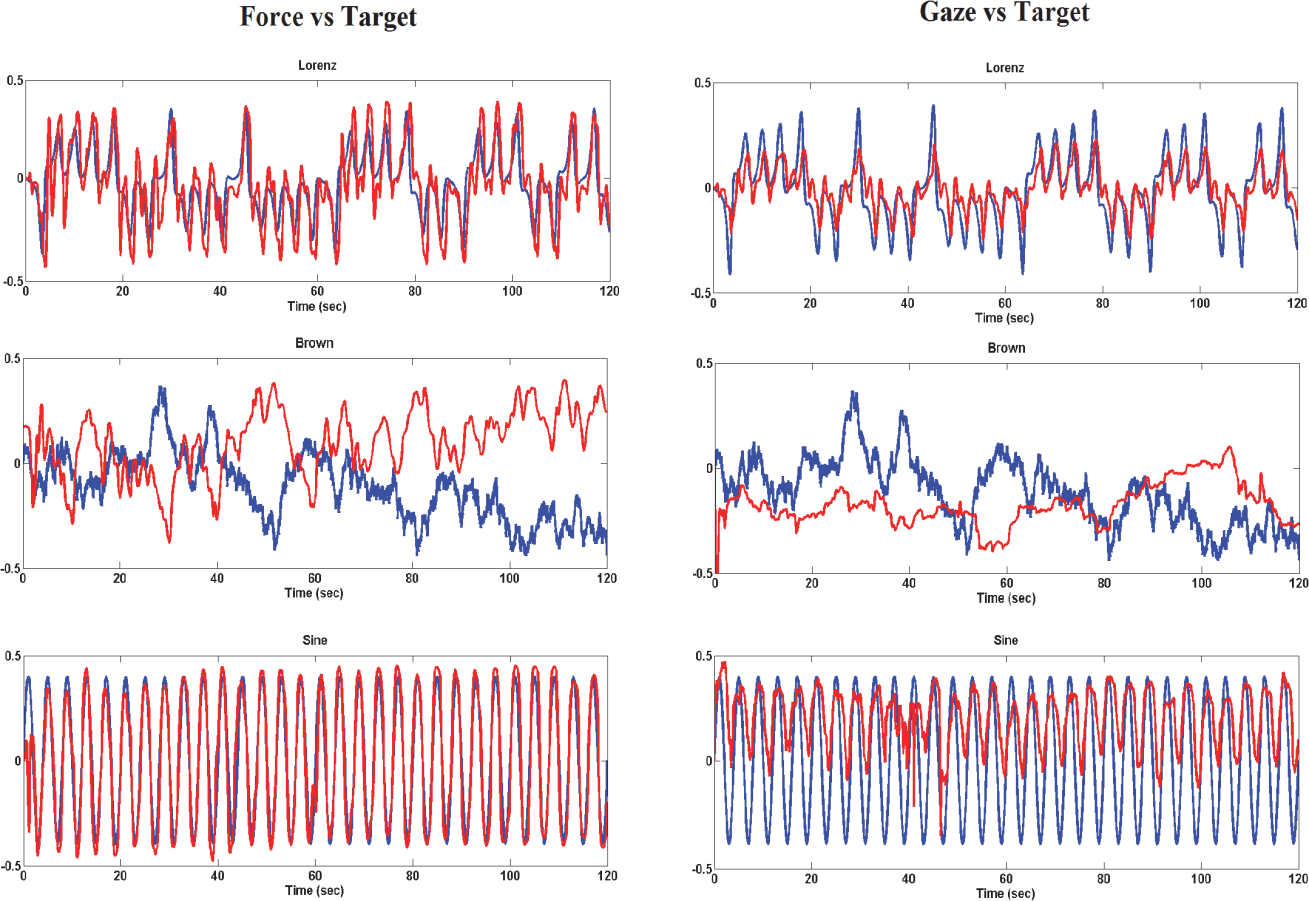
Performance-target curves. Performance (red line)–target (blue line) curves plotted for the three stimuli motions of a representative trial

Fig. 3 shows exemplar performance (force and gaze) curves superimposed on the target waveforms for a representative trial of one participant. With the exception of the brown noise stimulus motion, participants could entrain their postural sway and gaze to the motion of the visual stimulus reasonably well when sway and gaze were guided by the sinusoidal and Lorenz attractor stimulus motion.

### Coherence

#### Posture

**Fig 4:**
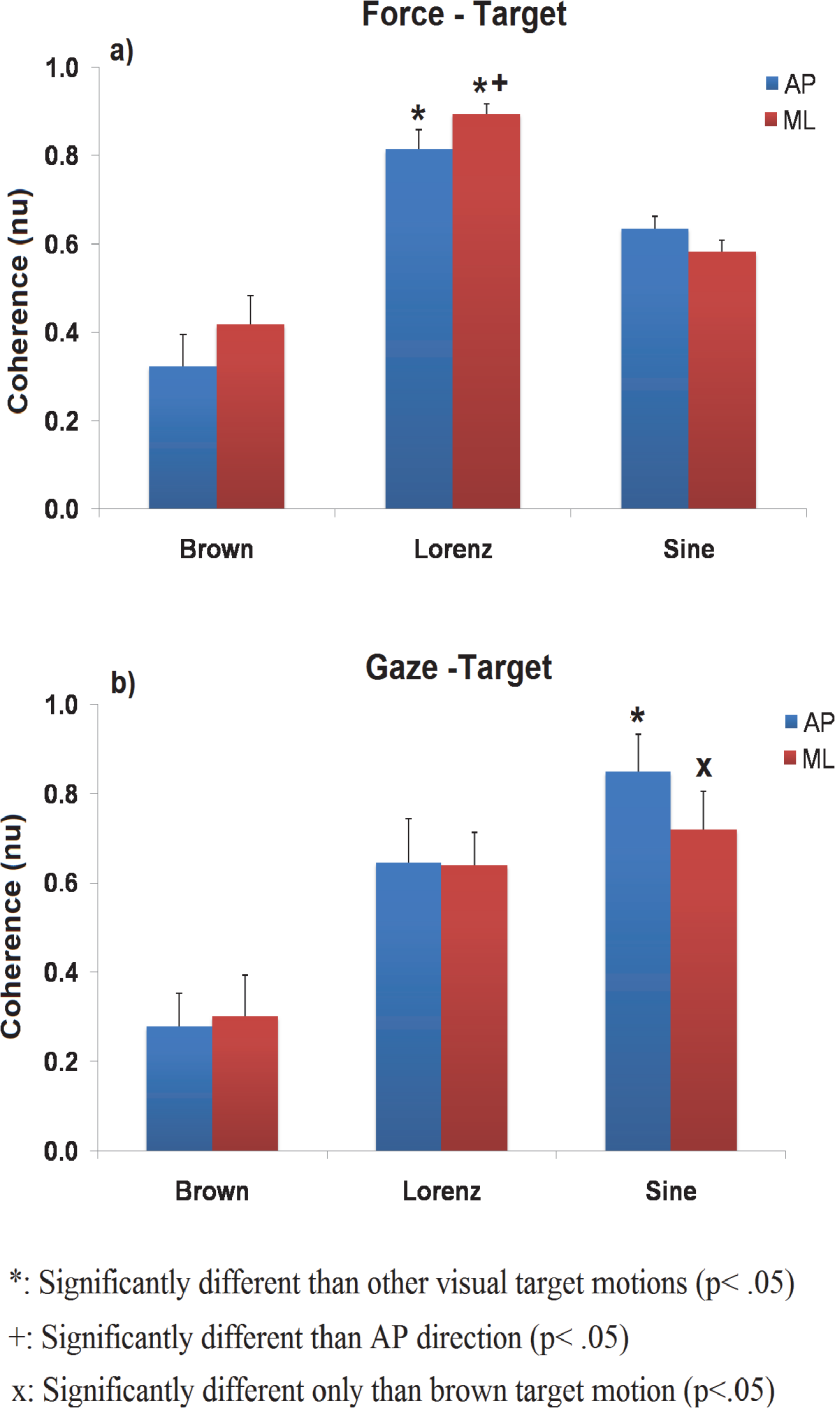
Coherence results. Force-target (a) and gaze-target (b) coherence for different stimuli motions and sway directions.

Group results for the force-target coherence are plotted in Fig. 4A. Analysis revealed a significant main effect of stimulus motion complexity on the magnitude of the coherence between the resultant force vector and the visual target signal (F (2, 18) = 46.769 p = .001). Post hoc pair wise comparisons between the three signals revealed that the magnitude of force-target coherence was significantly higher when postural sway was guided by the Lorenz attractor target motion than when was guided by the sinusoidal (AP, t(9) = 4.284, p = .002, ML, t(9) = 11,986, p = .001) and brown noise target motion (AP, t(9) = −7.860, p = .001, ML, t(9) = −8.369, p = .001). Moreover a significant stimulus complexity x direction interaction (F (2, 18) = 4.074, p = .035) revealed that differences between the two directions were dependent on the stimulus complexity. Specifically, post hoc pair wise comparisons between the two directions revealed that the magnitude of force-target coherence was significantly higher in the ML than the AP direction but only when sway was guided by the Lorenz attractor stimulus motion (t(9) = −3,159, p = .012).

#### Gaze

Group results for the gaze-target coherence are plotted in Fig. 4B. Analysis revealed that the magnitude of gaze-target coherence was depended on the complexity of the stimulus motion (F(2,18) = 23.147, p = .001). Specifically, both the sinusoidal and Lorenz attractor visual motions produced significantly higher gaze-target coherence than the brown noise motion in both directions (AP: (t(9) = −2.741, p = .023, (t(9) = −4.088, p = .003); ML: (t(9) = −4.056, p = .003),(t(9) = −4.840, p = .001)). However, in contrast to the force-target coupling, the magnitude of gaze-target coherence was higher when postural sway was guided by the sinusoidal than the Lorenz attractor visual motion (t(9) = −2.354, p = .043). This difference was significant only in AP and not in ML direction.

### Cross Approximate Entropy

#### Posture

**Fig 5:**
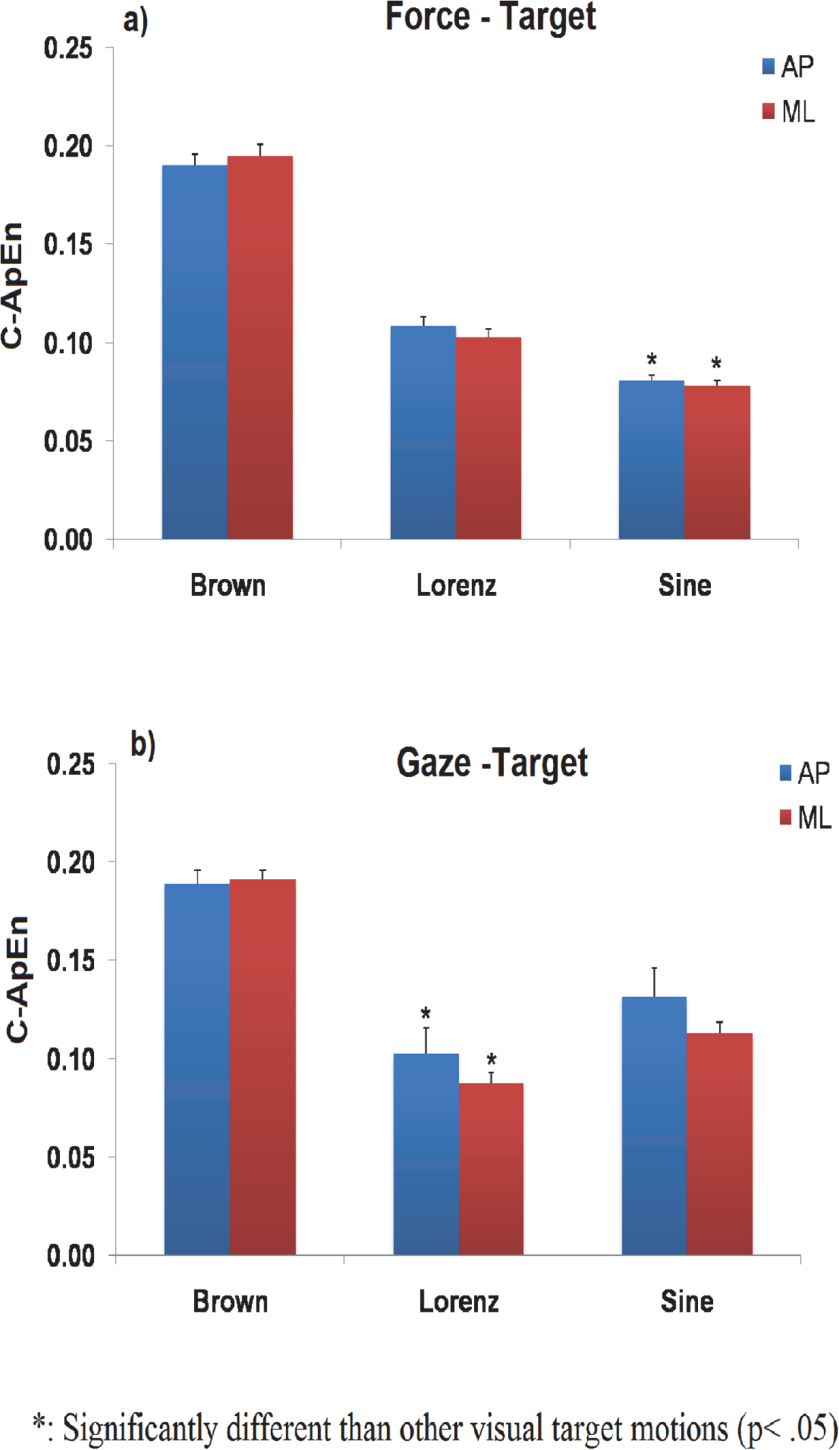
Cross Approximate Entropy results. Force-target (a) and gaze-target (b) Cross Approximate Entropy for different stimuli motions and sway directions.

Group results on the Cross ApEn metric are shown in Fig. 5. The Cross ApEn between force and target (Fig. 5A) was particularly sensitive to the complexity of the visual stimulus motion (F(2,18) = 210.713, p = .001). This was significantly higher when sway was guided by the brown noise visual motion than the other two target motions suggesting a greater degree of asynchrony between posture and target movements (AP: (t(9) = 12.570, p = .001, (t(9) = 18.316, p = .001); ML: (t(9) = 12.762, p = .001),(t(9) = 17.075, p = .001)). In addition, the Cross ApEn between the force and target signals was significantly lower when sway was guided by the sinusoidal than the Lorenz attractor visual motion revealing a more synchronous force-target coupling (AP, (t(9) = 5.369, p = .001); ML (t(9) = 5.692, p = .001). No significant direction effect on the force–target coupling was noted suggesting that the effect of target motion complexity was similar in both sway directions.

### Gaze

A significant main effect of visual motion complexity on the gaze-target Cross ApEn (F(2,18) = 95.031, p = .001)suggests that gaze was also sensitive to the visual stimuli motion complexity (Fig. 5B). Similarly to the force-target coupling, the Cross ApEn value was significantly higher when gaze was guided by the random (brown noise) than the sinusoidal and the Lorenz attractor stimulus motion ((AP: (t(9) = −7.236, p = .001, (t(9) = 3.628, p = .006); ML: (t(9) = 28.721, p = .001),(t(9) = 11.889, p = .001))). Interestingly however, the gaze-target Cross ApEn was significantly lower when gaze was guided by the Lorenz attractor than the sinusoidal visual stimulus motion (AP(t(9) = −2.659, p = .026); ML (t(9) = −3.722, p = .005) revealing a more synchronous eye tracking of the complex than the periodic visual target motion.

## Discussion

The novel finding of this study is that humans can track, with their gaze and posture, the complex motion of a visual target equally well or even better than the motion of a target moving periodically, regardless of whether tracking is performed in the AP or ML direction. This finding was confirmed by both metrics of performance-target coupling used in this study.

### Gaze performance

As expected, gaze showed a higher degree of coherence and lower Cross ApEn with the sinusoidal than the brown noise motion suggesting tighter coupling to the predictable target motion. Moreover, gaze synchronized better to the complex target motion as revealed by the lower Cross ApEn between gaze and the Lorenz attractor than the sinusoidal signal. Gaze in our task was defined by the direction of the visual axis (the line connecting the centre of the fovea and the point of fixation) in the two-dimensional coordinates of the projection screen. This reflected the eye and head movement in smoothly pursuing the target since the stimulus motion was sufficiently slow to eliminate the recruitment of eye saccades. The more synchronous gaze-target coupling when tracking the Lorenz attractor signal suggests that the complex target motion imposes a higher demand on attending to the visual target as opposed to the sinusoidal stimulus motion which is more predictable. Our findings complement previous evidence showing that the complexity of the visual stimulus elicits reciprocal complexity in the response of the smooth pursuit gaze behavior [14]. Eye-tracking literature connecting stimulus motion qualities to properties of smooth pursuit eye movements [24,25] confirms that humans tend to demonstrate complex gaze behavior, aligning with the idea that a search state with chaotic properties provides the visual system access to an optimal amount and quality of information. For this reason, studies investigating the relationship between environmental statistics and neural responses suggest that neural representation in the visual cortex is more efficient when visual scenes are natural, i.e. fractal [26]. The more efficient encoding of natural images or sounds assumes non-linear response properties of typical neurons in the primary visual cortex or auditory nerve [27].

Gaze-target coherence was still higher for the sinusoidal than the Lorenz attractor signal when the target was moving in the AP direction, whereas in the ML direction there was no difference between the two target motions. This difference in the AP direction could be due to the additional visuo-motor transformation required for tracking the vertically moving target and converting this information into forward-backward sway. The fact that gaze demonstrated a greater coupling to the sinusoidal than the Lorenz stimulus motion in the frequency domain may be attributed to the smaller amplitude of the gaze signal. As it is evident from Fig. 3, gaze is responding with significantly smaller amplitude against the sine stimulus signal in comparison to the Lorenz stimulus signal. Even though gaze was more synchronous (i.e. lower Cross ApEn) with the Lorenz stimulus motion it is possible that the organized complexity of the Lorenz attractor evoked a faster eye pursuing movement which resulted in a deviation of the gaze signal from the dominant sway frequency (0.25Hz) and therefore a lower gaze-target coupling to the Lorenz signal in the frequency domain.

### Postural performance

Force-target coupling was greater when the target was moving periodically than randomly (simulated by the brown noise signal). This finding complements previous evidence showing that a predictable target moving in the ML direction is tracked with a shorter time delay and higher accuracy than an unpredictable one [1]. Moreover, our results suggest that humans can entrain their posture to a complex visual motion, simulated by a Lorenz attractor signal, equally well or even better than to a periodic visual target motion. Tracking of a moving target with the whole body requires the coordination of the body’s multiple degrees of freedom in order to convert joint to spatial coordinates through a single point of force application. An additional transformation of the projector screen coordinates (up-down) to the body’s spatial coordinates (forward-backward) was required for AP tracking. The complexity of the sensory-motor transformations underlying this task may explain why entrainment was greater to the complex than the periodic target motion. This is because tracking of a complex stimulus motion allows for more flexibility and better resembles the inherent variability characterizing healthy human postural behavior.

Postural sway synchronized better with the periodic than the more complex target motion as indicated by the lower force-target Cross ApEn for the sinusoidal than the Lorenz attractor visual motion. The fact that posture showed a more synchronous coupling with the sinusoidal stimulus motion suggests that participants were able to predict the target motion and adjust their posture employing an open loop type of control without necessarily attending to the stimulus when tracking the sine wave. Our results are in agreement with experimental evidence from the tapping literature suggesting that synchronizing with biologically variable auditory rhythms involves similar internal processes as with other variable rhythms (whether totally random or comprising lawful regularities), but different from those involved with a regular metronome [28,29]. Specifically, synchronizing to a regular (periodic) metronome is assumed to involve an internal timekeeper prescribing time intervals in an open-loop manner and being completed by an error-correction process to achieve synchronization [30,31]. On the other hand, synchronizing to a biological rhythm is assumed to involve strong anticipatory processes [13] for short-term prediction and correction of periods and/or asynchronies. Instead of prediction on a local time scale however, strong anticipation assumes coordination on a non-local time scale. Therefore, based on the strong anticipation principle, entraining to a Lorenz attractor derived signal would require the coordination of posture to the global temporal structure of the stimulus signal and not on the local temporal changes. In other words, the anticipatory control of posture during tracking of the Lorenz attractor would depend not only on the past and current states of the system but also on future states. [15,16]. Indeed, the complex stimulus motion required the continuous pursuing/attendance of the visual target motion, which explains the greater temporal gaze-target coupling. Although, manual tracking literature suggests a common drive (or motor plan) to both eye and hand tracking systems [32], this may not be true for posture because of the more abstract cortical representation and multiple degrees of freedom involved in postural control.

### Effect of direction

Entrainment of posture and gaze to the target motions of varying complexity was similar regardless of whether tracking was performed in the AP or ML direction. Of note here is the fact that during the experiment, special attention was given to maintain a consistent stance width across all trials performed in both directions. AP tracking was expected to result in greater entrainment than tracking in the ML direction because visual information about self-motion and orientation is available predominantly in the AP [33,34]and to a lesser extent in the ML direction[35]. Nevertheless, tracking of a target moving in the vertical direction by voluntarily swaying in the sagittal plane required an additional coordinate transformation between the target’s screen (up-down) and the body’s spatial (forward-backward) coordinates which increased the computational load of the tracking task. For ML target tracking on the other hand, the direction of the visual stimulus motion was congruent with the postural sway direction and therefore did not require any additional transformation. The absence of differences in performance-target coupling between AP and ML postural tracking suggests that the two effects, the greater AP entrainment on one hand and the additional computational load of transforming body coordinates to screen coordinates on the other were canceled out. Several issues however are prone to further investigation. First, the greater entrainment to visual stimuli motion in the AP compared to the ML direction has been reported for unintentional looking and standing and not for voluntary intentional postural tracking. It is possible that visual stimuli motions are differently perceived and processed in quiet standing and voluntary postural tracking because the two tasks involve different visuo-motor transformations [15]. Second, for both AP and ML tracking the target motion was congruent to the body and gaze motion and intuitively selected. Whether similar results could be obtained for incongruent target-performance directions (e.g. visual stimulus moving in the AP direction while tracking is performed in the ML direction) remains to be seen in future studies.

### Conclusions and practical considerations

The results of the present study revealed that humans can track, with their gaze and posture, the complex motion of the visual stimulus equally well or even better than the motion of a target moving predictably. This novel finding complements recent multidisciplinary research proposing that information exchange is improved between complex long-range correlated systems [36]. Healthy physiological movement and posture in particular exhibits a natural organized complexity (chaos) whereas loss of complexity is indicative of aging, disease and pathology [10,37,38]. The optimization of information exchange by complexity matching between the human body and environmental stimuli reveals the importance of cooperative interactions over many possible perturbation timescales: a multiscale complexity that enhances system adaptability and learning [7]. Practically, the newly acquired visuo-motor transformation that goes together with previously learned responses is changed by the introduction of new critical stimuli without the presence of invariance in memory. Such a pattern is the result of effective dynamic neural interactions in sensory cortex and not of “purifying” stimuli. Thus, the process is dynamic and not just noisy involving active nonlinear cooperation between components and not just information processing.

In sum, based on our findings we propose that tracking of visual cues of a higher degree of complexity could be ecologically more sound and a better type of stimulus for optimizing visuo-motor learning while maintaining functional variability in the motor output. This type of practice may enable the human system maintain and use its functional degrees of freedom and adaptability. In this context, further studies should be designed to assess the sensitivity of the human perceptual-motor systems to the (long-range correlated) structure of fluctuations in environmental stimuli. This investigation may contribute to optimize human/environment interactions in several contexts, as the use of rhythmic auditory stimulation for gait rehabilitation in Parkinson’s patients [39]. Despite the potential that this emerging field presents for a) improving our understanding of dysfunction, b) providing sensitive biomarkers for evaluating interventions, and c) designing novel interventions based on restoration of complexity, its actual impact in the field of rehabilitative medicine has been minimal. The present study is an effort towards addressing this knowledge gap.

## Supporting Information

S1 Dataset Data file of all subjects. Coherence and Cross Approximate Entropy data for all subjects.
(XLSX)
